# A Holistic Assessment of Polyethylene Fiber Ingestion in Larval and Juvenile Japanese Medaka Fish

**DOI:** 10.3389/fphys.2021.668645

**Published:** 2021-08-04

**Authors:** Elizabeth DiBona, Lee J. Pinnell, Annika Heising-Huang, Simon Geist, Jeffrey W. Turner, Frauke Seemann

**Affiliations:** ^1^Department of Life Sciences, Texas A&M University-Corpus Christi, Corpus Christi, TX, United States; ^2^Institut für Genetik, Technische Universität Braunschweig, Braunschweig, Germany; ^3^Center for Coastal and Marine Studies, Texas A&M University-Corpus Christi, Corpus Christi, TX, United States

**Keywords:** microplastic, pollution, fish, toxicology, fiber, development

## Abstract

Microplastic pollution is of public concern for global environmental health, aquaculture, and fisheries. Toxicity studies have shown that microplastic ingestion may cause intestinal damage, microbiota dysbiosis, and disturb the lipid and energy metabolism in fish. To determine the impact of environmentally relevant, chronic, low dose microplastic fibers on fish health, medaka larvae, and juveniles were exposed to five concentrations of polyethylene (PE) fibers for 21 days through the feed. Fish growth and condition were assessed to determine the overall impact on fish health. To identify impaired energy intake, the gastrointestinal tract (GIT) integrity was evaluated at the molecular and cellular levels. Microbiota analysis was performed by comparing the top seven most abundant phyla present in both larval and juvenile fish exposed to 0, 1.5, and 3 PE fibers/fish/day. A shift in the phyla Proteobacteria and Bacteroidetes were observed. Larval samples demonstrated decreased proteobacteria abundance, while juvenile samples displayed an increase in abundance. Relative gene expression of key digestive genes from GIT tissue was quantified using real time-quantitative polymerase chain reaction. An effect on digestive gene expression potentially affecting nutrient absorption and antioxidant production was indicated *via* a significant decrease of solute carrier family 6 member 6 expression in larvae exposed to 6 fibers/fish/day. No significant molecular changes were observed in juvenile GIT tissue, although a non-monotonous dose-response was observed. GIT morphology was analyzed using histomorphological observations of the GIT mucus and cell types. No significant impairment of the GIT epithelial layers was observed in larvae or juveniles. To assess growth and condition, Fulton’s condition factor was measured. No differences were observed in larval or juvenile growth. Comparisons of different developmental stages allowed for identifying vulnerable developmental stages for microplastic exposure; larvae were more susceptible to molecular changes, while shifts in juvenile microbial communities were similar to changes reported post-polystyrene microplastic sphere exposure. This study is one of the first to provide toxicological data on the risk of PE fiber ingestion during fish development stages. Results indicate no imminent threat to fish condition at current measured environmental levels of microplastics; however, close monitoring of vital spawning grounds for commercially important fishes is recommended.

## Introduction

Awareness of widespread plastic pollution in marine, freshwater, and terrestrial ecosystems is a worldwide environmental issue. Over 220 wild species have been found to consume microplastics (Cole et al., 2011; Lusher et al., 2017). Microplastics (plastics particles < 5 mm) are the most numerous form of marine debris found in the environment, with fibers and fragments being the most abundant shapes of ingested microplastics (de Sa et al., 2018). Fibers are mainly sourced from the degradation of macroplastics and textile fiber shedding from domestic washing with polyethylene (PE) being the most common microplastic type found (Pirc et al., 2016; de Sa et al., 2018). The location of microplastics in the aquatic environment depends on their chemical composition and density, which directly affects the potential of marine organisms’ exposure to these contaminants.

Many different types of aquatic organisms, including teleost fish, mussels, and zooplankton, ingest microplastics inadvertently while feeding (Browne et al., 2008; Cole et al., 2013; Deudero and Alomar, 2015; Romeo et al., 2015; Granby et al., 2018). Ingestion of microplastic can harm the GIT mechanically and lead to injury and tissue structure alteration (Peda et al., 2016; Law, 2017). Various forms of physical damage in fish have been attributed to microplastic ingestion, such as intestinal lesions, dead tissue, and pro-inflammatory response (Ahrendt et al., 2020; Solomando et al., 2020; Zhang et al., 2020). Microplastic ingestion has also been observed to affect digestive function, immune response, and microbiota dysbiosis (Huang et al., 2020). Different polystyrene microplastic shapes caused intestinal damage and malfunction, specifically microbiota dysbiosis (Jin et al., 2018; Lu et al., 2018; Qiao et al., 2019; Feng et al., 2020). One recent study, sequencing the V3-V4 region of the 16S rRNA gene, revealed changes at the phylum level in colon microbiota of mice; both Proteobacteria and Actinobacteria abundances increased in the microplastic exposure groups (Lu et al., 2018). Microbiota communities can affect the interaction of the GIT and multiple body systems, e.g., brain, endocrine and immune system and modify feeding behavior, digestion, and metabolism (Butt and Volkoff, 2019). Identification of a shift in the microbiota community of exposed medaka will provide insight into effects on digestion and metabolism. Metagenomic sequencing of the V4 region of the 16S rRNA gene will provide information on the taxonomic composition of the microbiota within medaka GITs (Costa and Weese, 2018).

Moreover, significant changes on the molecular level indicative of oxidative stress and inflammation have been evidenced in aquatic organisms exposed to microplastics (Cole et al., 2011; Choi et al., 2018). Measuring changes in expression of key genes found in the GIT can identify the potential impacts PE fiber has on fish nutritional competence and overall GIT function. The digestive hormones glucagon-like peptide 1 (GLP) and peptide YY (PYY) is secreted by intestinal epithelial endocrine L-cells and regulate food intake (Rønnestad et al., 2013). GLP increases glucose availability through glycogenolysis (Holst, 2007; Polakof et al., 2012). PYY has been shown to decrease gastric and pancreatic secretions (Batterham and Bloom, 2003; Holzer et al., 2012; Polakof et al., 2012). Insulin (ISN), as the third hormone released by the pancreas, promotes critical nutrient uptake and stimulates energy reserves (Smith, 1980; Nelson and Sheridan, 2006). Trypsinogen (TRP), representing the proteolytic enzymes, is the enzyme trypsin’s precursor and is necessary for protein digestion (Nelson and Sheridan, 2006; Rønnestad et al., 2013). Upon release, TRP is activated in the fish’s GIT and becomes the digestive enzyme trypsin, which is vital for the digestion of proteins and has been found to have a positive relationship with growth (Smith, 1980; Rungruangsak-Torrissen et al., 2006). Like the taurine transporter solute carrier family 6 member 6 (slc6a6), nutrient membrane transporters are found in the plasma membrane throughout the GIT. Slc6a6 is required for amino acids and glucose uptake and plays a role in the Nrf2 pathway, which induces antioxidant production (Smith, 1980; Hybertson et al., 2011; Rønnestad et al., 2013). Deregulated expression of these genes can provide an insight into feeding, digestion, and nutrient uptake post microplastic exposure. Together, mechanical damage, microbiota community, and molecular assessments can give insight into the impact microplastics have on organism growth and overall health (Masura et al., 2015; Sussarellu et al., 2016; Beiras et al., 2018; Pannetier et al., 2020).

The majority of studies have focused on the impacts of polystyrene microplastic spheres on mature organisms using exposure concentrations 2–7 times higher than concentrations currently observed in the environment (Lenz et al., 2016). To further knowledge surrounding the impacts of microplastic environmental pollution, potentially susceptible developmental stages of fish inhabiting contaminated bays and estuaries should be assessed utilizing the more commonly found PE fibers.

Employing a chronic exposure scenario to PE fibers at concentrations similar to those found in previous research sampling both marine and freshwater environments, the potential impacts of PE fiber ingestion on microbiota composition, molecular gene expression, tissue integrity, and growth and condition are evaluated in larvae and juvenile Japanese Medaka fish (*Oryzias latipes*) in the present study (Lusher et al., 2013; Beer et al., 2018; de Sa et al., 2018). The GIT integrity, growth, and condition are critical indicators of fish health. Chronic exposure to environmentally relevant PE fiber concentrations is predicted to negatively affect growth and condition in a dose- and age-dependent manner. It is hypothesized that oral uptake of PE fibers targets the gastrointestinal tract (GIT). Ingestion of microplastics can disturb the GIT epithelial layer and increase inflammation in the GIT. Furthermore, we hypothesize that impacts will be life-stage dependent, and higher doses of PE fibers are expected to induce more drastic effects. Molecular deregulation of nutrient uptake pathways can increase GIT inflammation and change the GIT mucus layers affecting nutrient uptake, altering the fish’s energy budget, and potentially leading to reduced fish health.

## Materials and Methods

### Exposure Experiment

#### Microplastic Fiber Preparation

**FIGURE 1:**
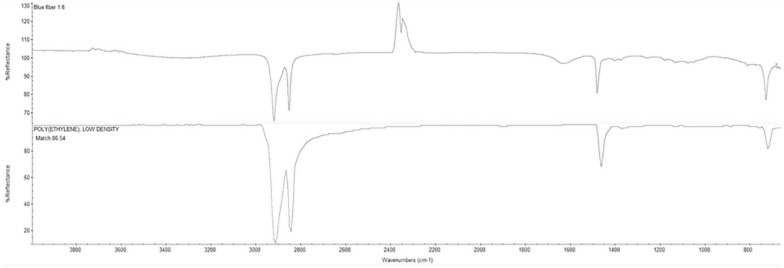
Fourier Transform Infrared - Attenuated Total Reflectance (FTIR-ATR) results **(upper spectrum)** for blue PE multifilament yarn from Lumat (United States) revealed an 86% match to standard polyethylene low density signal from the XYZ database **(lower spectrum)**.

Polyethylene fibers were chosen due to their commonality and abundance among microplastics found in wild specimens. A microplastic:fish size ratio of 1:4 was observed by Hajovsky and Geist (pers. communication) while assessing microplastic ingestion of juvenile fish in Corpus Christi Bay, Texas. Blue PE multifilament yarn was provided by Lumat (United States) (Supplementary Figure 1). The plastic material was received and analyzed using an FTIR-ATR (Thermo Fisher Scientific) and identified as an 86% match to PE low-density material (Figure 1). These PE fibers were observed to sink, but not to cluster, and thus, aeration was added to each tank to prevent sinking and allow fibers to be distributed within the water column during exposure. A microtome was used to cut the PE fibers into 100 μm increments (Cole et al., 2016). The PE multifilament yarn was folded and cut into approximately 9 mm long sections. The sections were then embedded in paraffin and cut into 100 μm sections. The paraffin film from the microplastic fibers was removed by overnight clearing (HistoChoice^®^ clearing agent). Fibers were then washed and filtered using DI water onto a cellulose nitrate filter. Microplastic fibers were stored on the filter in a closed glass petri dish until later. To obtain 400 μm sections of the multifilament yarn, a paper cutter and ruler were used to cut microplastic into the appropriate length. The sections were then placed on the cellulose nitrate filter and stored in a closed glass petri dish until later use. The respective PE fiber lengths were chosen according to the 4:1 fish size/fiber length ratio reported in fish field samples from the Gulf of Mexico (Hajovsky, 2019).

#### Model Organism

Japanese medaka, *Oryzias latipes* (*OL*) orange-red inbred strain established at TAMU-CC in 2018 were used as a model for this study (IACUC # 19-03). Larval and juvenile age groups were selected based on development stage and potential vulnerability to microplastic ingestion: 7 days post-hatching (dph) represents the period of mouth opening and beginning of feeding for medaka larvae, and 1-month post-hatching presents a critical period for organ maturation and body growth for medaka (Kinoshita et al., 2009). Larvae at 7 dph (total length (TL): 0.04 ± 0.02 cm) and 1-month old juveniles (28 dph; total length (TL): 0.16 ± 0.04 cm) were randomly allocated to 2 L tanks at a density of 50 individuals per tank (*n* = 5). Water quality was maintained *via* weekly water changes (100%). Tanks were maintained at a temperature range of 25 ± 1°C, 12-h photoperiod, dissolved oxygen at 6 mg/per liter, nitrates below 20 mg per liter, nitrites below 0.1 mg per liter, ammonia below 0.01 mg per liter, and pH range from 7.7 to 8.2. Tanks were provided aeration to keep microplastic fibers circulating in the water column and to prevent settling of PE fibers on the bottom of the tank. Tanks were covered to prevent potential cross-contamination of microplastic fibers. All animal experimental procedures have been approved by the institutional animal care and use committee #19-05.

#### Larval and Juvenile Exposure

Larval and juvenile *OL* were exposed to five concentrations of 100 μm (larvae) and 400 μm (juvenile) long PE fibers, respectively. The PE fibers were added to approximately 0.1 g of ground dry larval feed and stored in 0.2 ml sterile PCR tubes before the exposure experiment. The daily dose of PE fibers was administered to larvae through two feedings for a 21-day chronic exposure period. The environmentally relevant exposure concentrations were 0, 0.5, 1.5, 3, and 6 PE fibers per individual per day for both age groups, with 5 independent replicate tanks holding 50 individuals per replicate. Upon completing the experiment, individuals were sacrificed through hypothermic shock, weighed, measured, dissected for tissue samples, and preserved for subsequent analyses.

### Microplastic Fiber Long-Term Retention

To assess retention of microplastic fibers in the GIT, 10 larvae and juvenile medaka per replicate (*n* = 5 pools of 10 individuals) were sacrificed 12 h after the last microplastic feeding *via* hypothermic shock and stored at -20°C until assessment. Fish were weighed, measured and the intestine was dissected out. PE fibers per individual GIT were counted with a stereomicroscope for each exposure concentration and the control.

### Microbiota Assessment

The microbiota composition was analyzed to determine if microplastic consumption at these concentrations caused dysbiosis. GITs of both larvae (*n* = 5 pools of 1-2 individuals per concentration) and juveniles (*n* = 5 pools of 1-2 individuals per concentration) medaka were collected post exposure from the exposure groups dosed with 0, 1.5, and 3 PE fibers per fish per day. These concentrations (0, 1.5, and 3 fibers/fish/day) were chosen based on preliminary reproduction assessment data indicating these groups may be more at risk (DiBona, 2020). DNA was extracted from the samples using a QIAamp^®^ PowerFecal^®^ Pro DNA Kit from QIAGEN. The V4 region of the bacterial and archaeal 16S rRNA gene was amplified using 515f/806rB primer constructs (Walters et al., 2016). The constructs contained Illumina specific adapters followed by 12 bp Golay barcodes on each forward primer. PCR was performed in replicate reactions containing 12.5 μL Phusion Hot–Start Flex 2X MasterMix (New England Biolabs, Ipswich, MA, United States), 0.2 μM final concentration of forward and reverse primers, 2 μL template, and nuclease-free water to equal 25 μL. Mock microbial community DNA standards (Zymo Research, Irvine, CA, United States) and no template controls were prepared with each PCR replicate. Amplification conditions were 98°C for 30 s followed by 30 cycles of 10 s at 98°C, 30 s at 55°C, and 1 min at 72°C. Final extension occurred at 72°C for 5 min. 25 μL from each amplicon library was then cleaned and normalized using the SequalPrep Normalization Plate Kit (Applied Biosystems, Foster City, CA, United States), and equal volumes of each normalized library were pooled. The pooled library was quantified using a Qubit 3.0 fluorometer and dsDNA High Sensitivity Assay Kit (Life Technologies, Carlsbad, CA, United States). The molarity of the pooled library was calculated and was diluted to a loading concentration of 6 pM. The diluted, pooled library was sequenced on an Illumina MiSeq instrument using paired-end chemistry (2 × 250 bp) with the addition of a 10% PhiX Control Library (Illumina, San Diego, CA, United States) to increase sequence diversity at the Shedd Aquarium’s Molecular and Microbial Ecology Lab (Chicago, IL, United States).

Raw sequence reads were processed using a combination of QIIME2 and phyloseq (Caporaso et al., 2010; McMurdie and Holmes, 2013). Reads were demultiplexed and checked for quality using QIIME2. Due to low-quality scores, reverse reads were omitted from further processing and samples were processed as single-end reads using forward reads only. DADA2 was used to filter reads for quality, remove chimeric sequences, and generate amplicon sequence variants (ASVs) within QIIME2 using a trim length of 242 bp (Callahan et al., 2016). Taxonomy was assigned using a Naïve Bayes classifier trained on the SILVA 132 release 99% OTUs database, where sequences had been trimmed to include only the 250 bases from the V4 region bound by the 515F/806R primer pair (Quast et al., 2013). Reads that mapped to chloroplast and mitochondrial sequences were filtered from the sequence variants table using the “filter taxa” function, and a phylogenetic tree was then generated using the “q2-phylogeny” pipeline with default settings, which was used to calculate phylogeny-based diversity metrics. Data were then imported into phyloseq using the “import_biom,” and “import_qiime_sample_data” functions and merged into a phyloseq object (McMurdie and Holmes, 2013). Alpha diversity was measured using the number of observed ASVs, Shannon’s diversity, and Faith’s phylogenetic distance. Samples were then proportionally transformed to a normalized read count equal to the lowest per sample read depth (12,412). Beta diversity was analyzed using UniFrac distances calculated using the R packaged “GUniFrac” (Lozupone et al., 2011; Chen et al., 2012). These distances were ordinated and plotted using phyloseq. Further, the relative abundances of ASVs within each sample were calculated and plotted using phyloseq.

### Digestive Gene Expression

Gastrointestinal tracts from larvae (*n* = 5 pools of 5 individuals per concentration) and juvenile (*n* = 5 pools of 5 individuals per concentration) medaka were isolated during dissection and stored at -80°C. mRNA was extracted from the tissue samples using 500 μl TRI-Reagent following the extraction protocol. Tissues were homogenized in 2 ml Precellys tubes with ceramic beads. Samples were incubated at room temperature (23°C) for 5 min. 50 μl of chloroform was added followed by gentle mixing. After incubation for 10 min at room temperature, the samples were centrifuged at 12,000^∗^*g* for 10 min at 4°C. The aqueous phase containing RNA was transferred into a new RNAse/DNAse-free 1.5 ml microcentrifuge tube and precipitated with 250 μl isopropanol, vortexed for 10 s, and incubated at room temperature for 10 min. Samples were then centrifuged at 12,000^∗^*g* for 8 min at 23°C. The supernatant was discarded, and the pellet was resuspended using 500 μl of 75% molecular grade ethanol. The samples were centrifuged at 7,500^∗^*g* for 5 min at 23°C. The supernatant was removed, and samples air dried to remove any remaining ethanol. Once dry, each sample pellet was resuspended using 50 μl of RNAse/DNAse free sterile water and stored at −80°C.

RNA quality and concentration were determined through gel electrophoresis and the BioSpectrometer (Eppendorf). Reverse transcription was performed to obtain cDNA for RT-qPCR using the Promega Reverse Transcriptase kit following the manufacturer’s instructions. Briefly, the RNA, oligo primers, random primers, and nuclease-free water were combined in a 0.2 ml PCR tube and incubated at 70°C for 5 min. Then, the reaction mix containing the reaction buffer, MgCl2, PCR nucleotide mix, reverse transcriptase, and water was prepared as a master reaction mix for all samples. The preincubated samples were removed from 70°C and placed on ice for 5 min. Next, 15 μl of the master reaction mix was added to each sample tube and the reverse transcription was performed (annealing: 25°C for 5 min, extension: 42°C for up to an hour, inactivation of the reverse transcriptase at 70°C for 15 min). The cDNA samples were stored at −20°C.

Real time quantitative polymerase chain reaction was performed with *O. latipes* digestive gene primers (TRP, ISN, GLP, PYY, and slc6a6) and three reference genes (18S, EF1a, and RPL7) (Supplementary Table 1) using a 1:2 dilution of template cDNA. Reference genes were selected based on previous research indicating 18S, EF1a, and RPL7 are suitable for normalization in RT-qPCR (Zhang and Hu, 2007; Kozera and Rapacz, 2013). Reference gene stability was verified using RefFinder (BestKeeper, DeltaCT, NormFinder, and Genorm) (Xie et al., 2012). Relative gene expression was performed using real time quantitative polymerase chain reaction in a 96-well plate with 10 μl of master mix and 2.5 μl of template per well for a total volume of 12.5 μl per well. The plate was sealed with a plastic membrane, centrifuged and then run in the qPCR machine (QuantStudio3) for 40 cycles (95°C for 5 min, 95°C for 25 s, 60°C for 20 s, 72°C for 30 s, 78–80°C for 15 s) followed by a meltcurve (95°C for 15 s, 60°C for 60 s).

### Gastrointestinal Tract Histomorphometric Analyses

The histomorphology of the GIT epithelial layer will be assessed to obtain information on tissue integrity and inflammation post-PE fiber exposure. Larval and juvenile specimens were sacrificed *via* hypothermic shock and fixed in 4% formalin (5 individuals per replicate, *n* = 5 pools of 5 individuals). Whole fish were embedded in 1.5% Agarose gels, dehydrated and embedded in paraffin. Serial cuts of the GIT of the medaka were adhered to microscope slides and left to dry overnight. Slides were stained with either Hematoxylin and Eosin stain (H&E) or Alcian Blue and Periodic Acid Schiff stain (AB-PAS) using an automated slide stainer (Thermo Fisher Scientific) and subsequently mounted with a glass coverslip using. Pictures were taken with cellSens Standard software on an Olympus BX53 compound microscope at a magnification of 40X. Image J software (Fiji) was employed for all image analyses (Schindelin et al., 2012).

**FIGURE 2:**
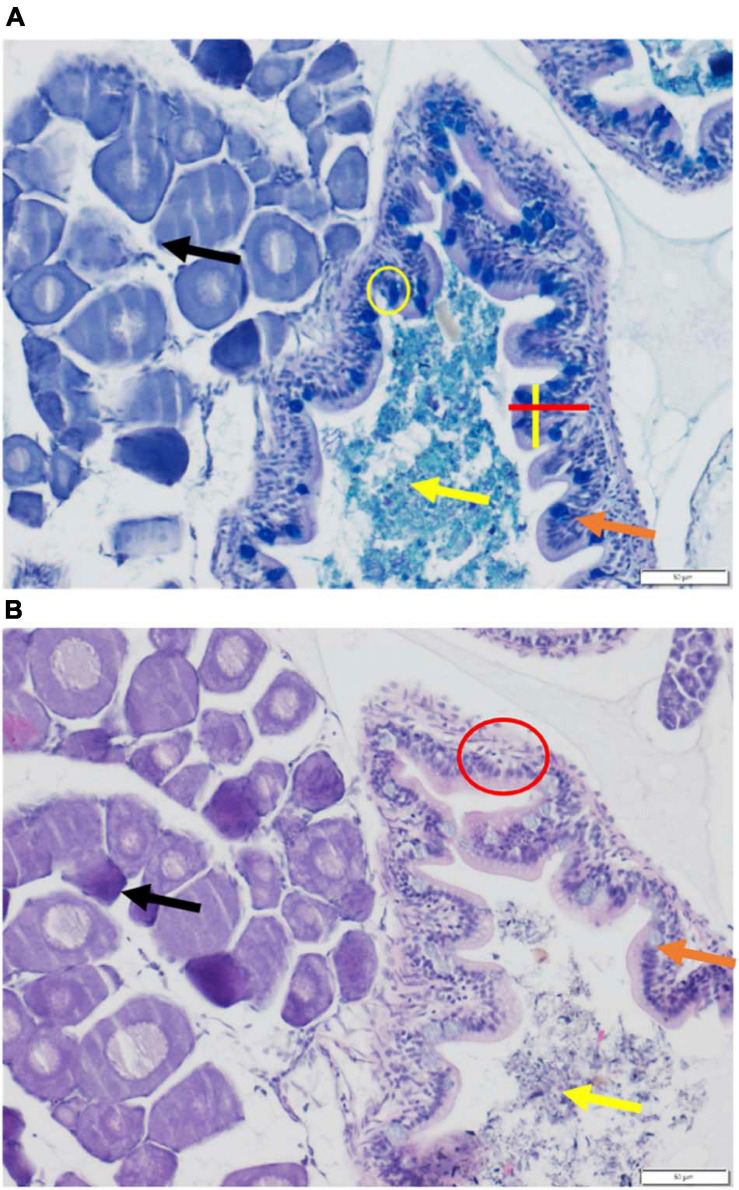
Stained histological sections of control (0 fibers/fish/day) larval medaka hindgut. **(A)** AB-PAS stained hindgut. Red line indicates a measurement of microvilli length. Yellow line indicates a measurement of microvilli width. Yellow circle encompasses goblet cell. Orange arrow indicates microvilli; yellow arrow indicates gut lumen/gut contents; black arrow indicates gonadal tissue. **(B)** H&E stained hindgut. Red circle encompasses example area assessed for leukocyte migration/inflammation. Orange arrow indicates microvilli; yellow arrow indicates gut/lumen contents; black arrow indicates gonadal tissue.

Leukocyte migration was assessed as an inflammation marker, affecting nutrient absorption (Smith, 1980). Inflammation was measured using H&E stain (Figure 2B). Sections from the hindgut of the fish were used for assessment; the hindgut was determined based on the presence of surrounding tissue such as gonadal tissue as well as the structure of the intestine (Mumford et al., 2007). Leukocytes counts were used to determine the degree of inflammation (Mumford et al., 2007). A score system of 0–4 was used to quantify the degree of inflammation (0 = no recruitment, 1 = minor recruitment (1–10 leukocytes), 2 = definite recruitment (11–20 leukocytes), 3 = significant inflammation (21–30 leukocytes), and 4 = severe inflammation (>31 leukocytes). An area from three sections of 5 individuals’ hindguts was assessed per concentration for a total of 15 sections per concentration. An area was defined to be 40 μm^2^, and areas were spaced by at least 20 μm to ensure no overlap. Assessed sections came from non-consecutive slides to ensure no duplicate counts or overlap.

AB-PAS staining allowed the assessment of mucus pH, goblet cell abundance and microvilli length and width (Figure 2A). GIT mucus is crucial for protecting epithelial cells and supporting digestion, and was assessed for pH, as pH fluctuations affect enzymatic activity (Hur et al., 2016; Sylvian et al., 2016). Mucus pH was measured using AB-PAS-stained slides as this stain allows for the differentiation of mucus layers and pH. Image J software (Fiji) was employed for the image analyses. Foregut sections were used for mucus assessment; foreguts were determined by the structure of the intestine as well as surrounding tissues. Assessed sections came from non-consecutive slides to ensure no duplicate counts or overlap. Mucus pH was determined based on a color scale using Image J software; images were converted to 8-bit grayscale and analyzed. On this gray scale 0 = pure black and 255 = pure white. An area of 40 μm^2^ from three sections of 5 individuals’ foreguts was assessed per concentration, for a total of 15 sections per concentration (*n* = 5 pools of 5 individuals).

Comparison of the mucus-producing goblet cell abundance allowed for assessment of PE fiber influences on mucus production and release (Smith, 1980; Hur et al., 2016). Goblet cell abundance was assessed using AB-PAS stained slides as this stain allows for the easy identification of goblet cells. Hindgut regions were used for goblet cell counts. Goblet cells were counted per microvilli. Microvilli were selected randomly from the slide and only considered if entire and intact. Three microvilli per section were assessed and then averaged to give a section count. Three sections per individual were assessed. Assessed sections came from non-consecutive slides to ensure no duplicate counts or overlap. Five individuals per concentration were assessed for a total of 15 sections per concentration (*n* = 5 pools of 5 individuals).

Microvilli length and width were assessed to determine if there is a variation in the overall surface area available for nutrient absorption (Smith, 1980). Microvilli length and width measurement were assessed using AB-PAS stained slides. Hindgut regions were used for microvilli length and width measurements. Microvilli length was taken perpendicular to the intestinal membrane. Microvilli widths were taken at the widest points of the microvilli. Microvilli were selected based on the microvilli selected for the goblet cell abundance measurement; microvilli counted for goblet cells were also measured for length and width. The same sections used for goblet cell abundance were used for microvilli length and width assessment. Three microvilli per section of individual were assessed and then averaged to give a section count. Three sections per individual were assessed. Five individuals per concentration were assessed for a total of 15 sections per concentration (*n* = 5 pools of 5 individuals).

### Condition Assessment

The assessment of growth and condition provide insights on the nutritional status and the fish’s overall health. Fulton’s condition factor (K) was measured to assess fish fitness and condition (Froese, 2006). Factor equation, $K=100\times \frac{W}{L^{3}}$, where K is the coefficient of condition factor, W is the body weight of the fish, and L is the standard length of the fish (Fulton, 1904). This index is a non-invasive biomarker used for the overall assessment of fish health. Each individual was measured before dissection (*n* = 5 pools of 45 individuals per replicate). A fish’s condition indicates the overall energy of the fish available for growth, feeding, reproduction, and other activities. With molecular and histomorphological analysis, the assessment of growth and condition will provide evidence of microplastic ingestion effects on fish health.

### Data Analysis

Unless otherwise specified, R Studio and R version 4.0.3 were used to analyze all data (R Core Team, 2020). Data are presented as means ± standard deviation. Data were tested for homogeneity of variances and normality using the Shapiro–Wilk’s method. All comparisons were made between the control group and the experimental groups. *P*-values < 0.05 were considered significant for all analysis. A one-way A nested analysis of variances (ANOVA) was used for analysis of RT-qPCR data (*n* = 5 replicates/pools of 5 individuals per concentration). If significant differences were present, a TukeyHSD post-hoc test was performed to identify significant differences. ANOVA was used for long-term retention assessment (*n* = 5 replicates/pools of 10 individuals per concentration), GIT histomorphometric analysis (*n* = 5 replicates/pools of 5 individuals per concentration), and fish condition assessment (*n* = 5 replicates/pools of 45 individuals per concentration). To identify differences of the GIT microbiota, pairwise Wilcoxon rank-sum tests were performed with a Benjamini and Hochberg correction (PW+BH) to control for false discovery rates during multiple comparisons (Benjamini and Hochberg, 1995). Pairwise permutational multivariate analysis of variance (PERMANOVA) using 9,999 permutations and a Benjamini and Hochberg correction (Benjamini and Hochberg, 1995) was used to test for significant differences in microbial community structure using the “vegan” (Oksanen et al., 2019) and “pairwiseAdonis” (Arbizu, 2017) packages. To ensure PERMANOVA results were not solely the result of unequal dispersion of variability between groups, permutational analyses of dispersion (PERMDISP) using 9,999 permutations were conducted for all PERMANOVA comparisons with the “vegan” package in R.

## Results

### Microplastic Fiber Long-Term Retention

**FIGURE 3:**
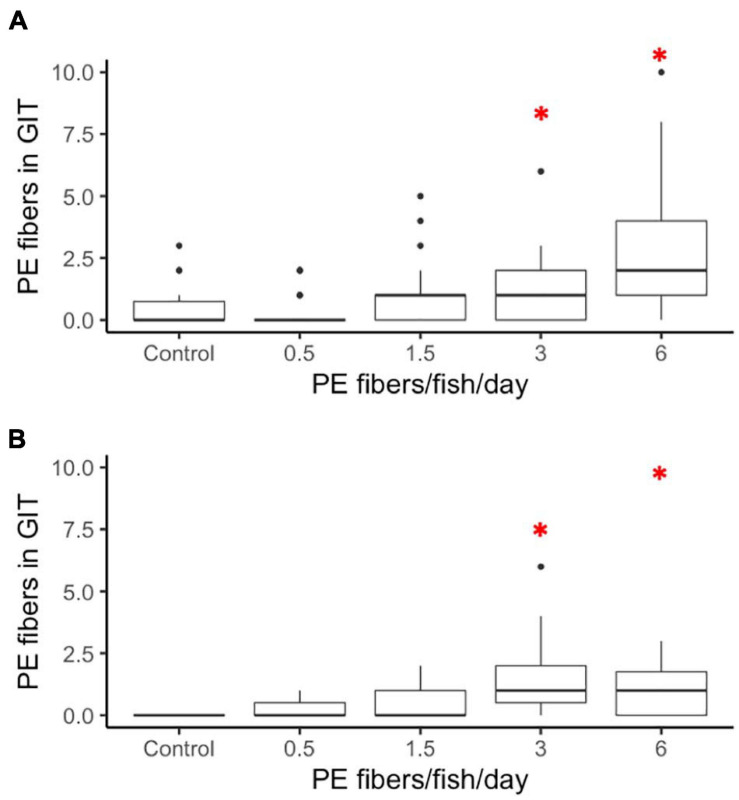
Long term retention of PE fibers in **(A)** larvae and **(B)** juveniles *O. latipes* after 21 days of exposure to control (0), 0.5, 1.5, 3, and 6 fibers/fish per day. Red stars indicate significant differences revealed by a nested ANOVA, ^∗^*p* < 0.05, *n* = 5.

In comparison to a 1-h short-term exposure retention experiment (Supplementary Figures 2, 3), chronic PE fiber exposure resulted in an extended fiber retention time in the GIT for both larval and juvenile medaka. Larvae exposed to both 3 fibers/fish/day (*p* = 0.0168) and 6 fibers/fish/day (*p* = 0.0000) retained significantly more fibers in the GIT compared to the control group (Figure 3A). The data indicated a non-significant increase of microplastic fibers in the GITs for 1.5 fibers/fish/day and 3 fibers/fish/day exposed larvae. In the juvenile group, microplastic fiber retention was significantly increased in individuals exposed to 3 fibers/fish/day (*p* = 0.0249) and 6 fibers/fish/day (*p* = 0.0249) (Figure 3B) compared to the control.

### Microbiota Analysis

**FIGURE 4:**
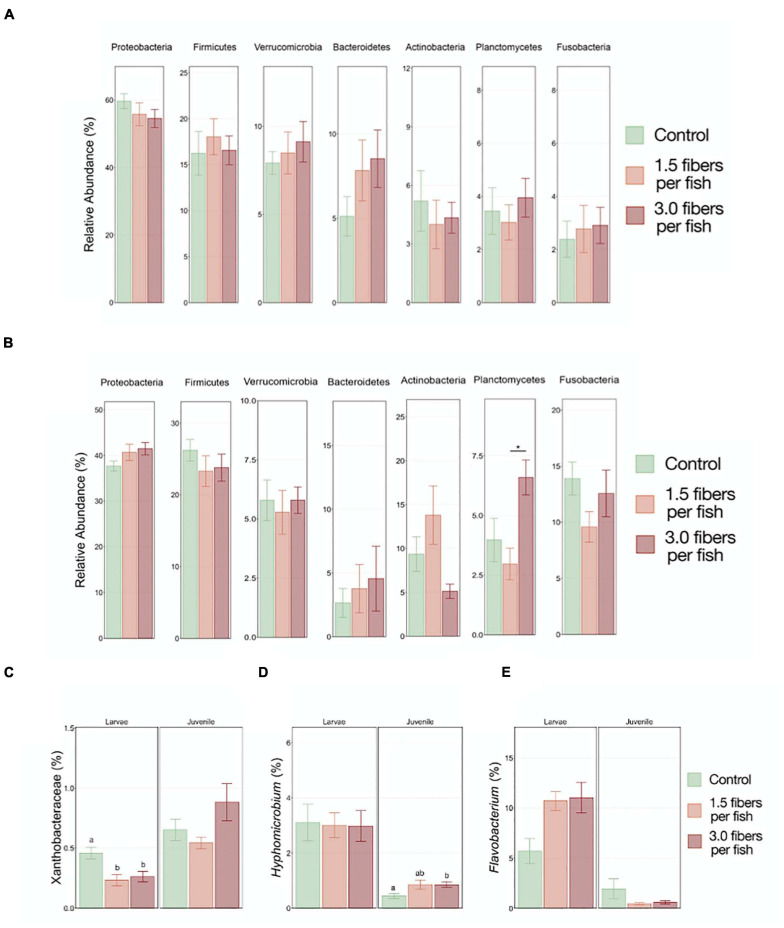
Comparison of the top seven most abundant gut microbiome phyla in larvae **(A)** and juvenile **(B)** *O. latipes* from the control, 1.5, and 3 fibers/fish/day exposure groups. Abundance of **(C)** Xanthobacteraceae (Proteobacteria), **(D)** Hyphomicrobium (Proteobacteria) and **(E)** *Flavobacterium* (Bacteroidetes) in larval and juvenile samples are depicted. Stars indicate statistically significant differences between treatment groups; different letters indicate statistically significant differences between control and treatment groups with (Kruskal–Wallis Test, *p* < 0.05; *n* = 4).

Microbiota analysis was performed by comparing the top seven most abundant phyla present in both larval and juvenile fish exposed to 0, 1.5, and 3 PE fibers/fish/day. Although no statistically significant differences found on the phyla level in either larval (Figure 4A) or juvenile fish (Figure 4B), a shift of the phyla Proteobacteria and Bacteroidetes was observed. Upon PE fiber exposures, proteobacteria abundance was either decreased (larvae) or increased (juveniles), which could be attributed to a significant reduction of the Xanthobacteraceae family in the larvae (Figure 4C) and a significant increase of the Hyphomicrobium family in the order Rhizobiales in juvenile fish exposed to 3 PE fibers/fish/day (Figure 4D). Within the Bacteroidetes phyla, an increase, although not statistically significant, of the genus *Flavobacterium* may account for the PE fiber exposure-induced increase on the phyla level in larval fish, but not in juveniles (Figure 4E). It is noteworthy, that non-monotonous dose-response patterns were observed in the GIT microbiota composition of juvenile fish for the phyla Planctomycetes, Fusobacteria, and Actinobacteria. PERMANOVA analysis assessing microbial community structures did not show any significant differences between the microplastic exposure concentrations and the control (Supplementary Figure 4).

### Impacts of PE Fiber Exposure on the Molecular Regulation of Nutrient Uptake

**FIGURE 5:**
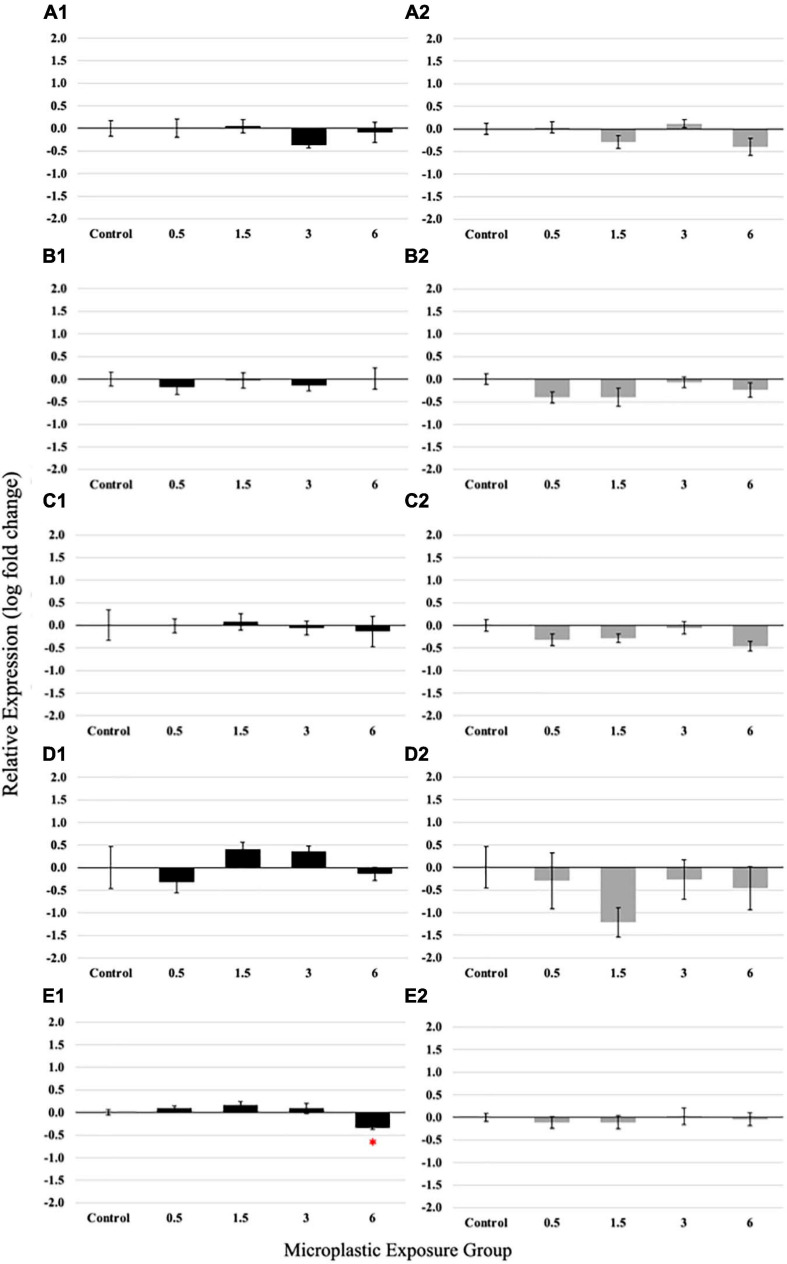
Relative expression of Glucagon **(A1)**, Peptide YY (PYY) **(B1)**, Trypsinogen (TRP) **(C1)**, Insulin (ISN) **(D1)**, and solute carrier family 6 member 6 (slc6a6) **(E1)** in larvae medaka after 21 days of exposure to 0 (control), 0.5, 1.5, 3, and 6 fibers/fish per day. Relative expression of Glucagon **(A2)**, Peptide YY **(B2)**, Trypsinogen **(C2)**, Insulin **(D2)**, and slc6a6 **(E2)** in juvenile medaka after 21 days of exposure to 0 (control), 0.5, 1.5, 3, and 6 fibers/fish per day. Data are presented as mean relative expression ± standard error (ANOVA, *p < 0.05, *n* = 5 replicates/pools of 5 individuals per concentration).

Polyethylene fiber-induced changes in the expression of selected molecular markers important for digestion and nutrient uptake may serve as early warning markers for adverse effects on the tissue and organism level. PE fiber exposure during the larval stage revealed non-monotonous dose responses of GLP and ISN (Figures 5A1,D1). For GLP, a reduced expression was observed in the 3 fibers/fish/day exposure concentration, while ISN expression was elevated for 1.5 and 3 fibers/fish/day exposure concentrations. Both expression levels returned to the control level at 6 fibers/fish/days exposure concentration; however, these trends were not statistically significant. The highest PE fiber concentration significantly decreased (*p* = 0.0151) the expression of slc6a6 (Figure 5E1). No significant differences were observed in the relative gene expression of PYY and TRP (Figures 5B1,C1).

Non-monotonous dose-responses were even more prominently observed in juvenile fish exposed to PE fibers (Figures 5A2–E2); again, however, these trends were not significant. Expression of GLP, PYY, TRP, ISN, and scl6a6 showed a consistent pattern of decreased expression in the 0.5 and 1.5 PE fiber/fish/day groups, a return to control levels in 3 fibers/fish/day exposed juveniles and approximately a two-fold change decrease of the measured gene expression in juveniles exposed to 6 PE fibers/fish/day.

### Impact of PE Fiber Exposure on the GIT Integrity

**FIGURE 6:**
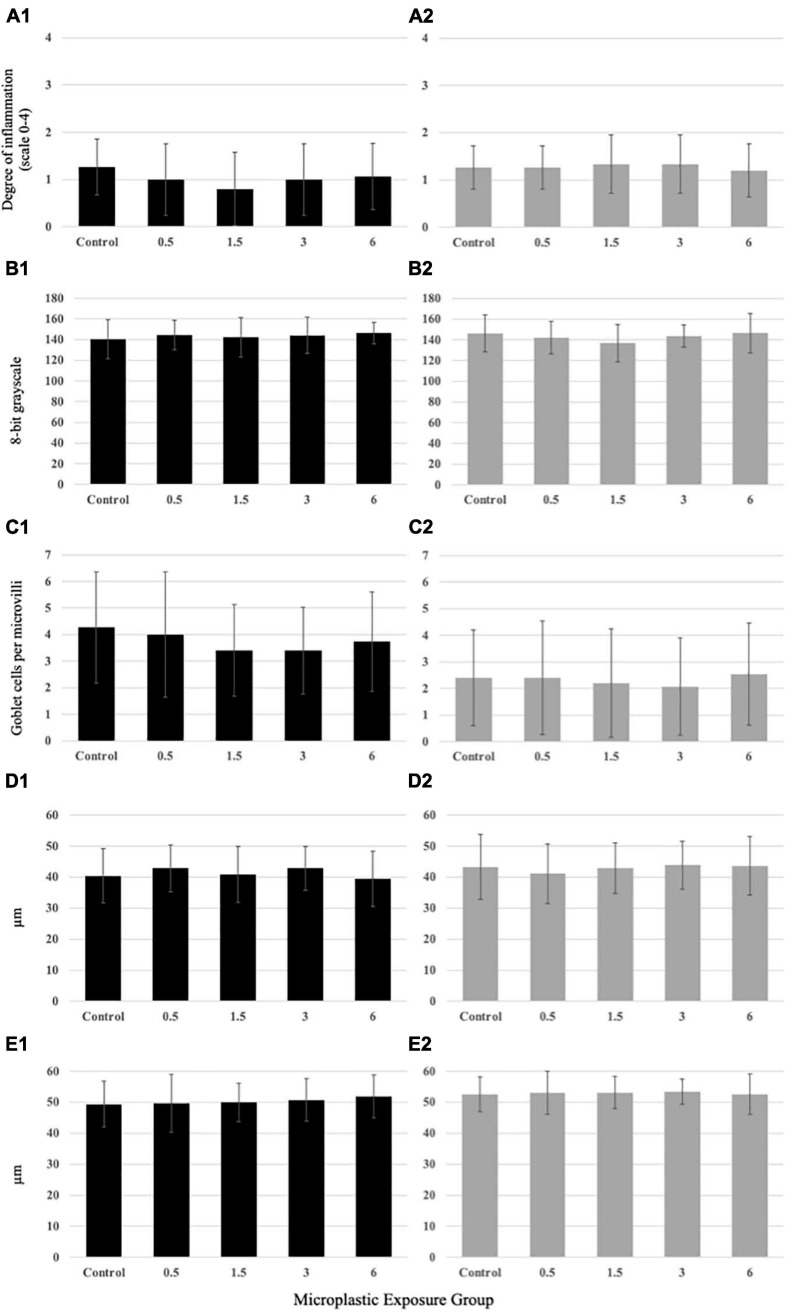
Histologicalanalysis results from larval **(A1–E1)** and juvenile**(A2–E2)** medaka exposed to PE fibers. No significantvariation at the larval or juvenile life stage among themicroplastic concentrations compared to the control for**(A1,2)** inflammation, **(B1,2)** mucus pH, **(C1,2)** goblet cell abundance, **(D1,2)** microvillilength, or **(E1,2)** microvilli width. Data are displayed asmean ± standard deviation (nested ANOVA, *p < 0.05, *n* = 5).

The PE fiber exposure regimes applied in this study did not induce inflammation of the hindgut of medaka larvae (Figure 6A1) or juveniles (Figure 6A2). All examined hindgut sections showed little to no leukocyte infiltration as indicator of inflammation. No significant changes in the mucus pH (Figures 6B1,B2), goblet cell abundance per microvilli (Figures 6C1,C2), and microvilli length and width (Figures 6D1,D2,E1,E2) were observed in either larvae or juveniles exposed to environmentally relevant levels of PE fibers. There were no variations in overall gut integrity for the larvae or juveniles (Supplementary Figures 5, 6).

### Assessment of Overall Fish Condition

**FIGURE 7:**
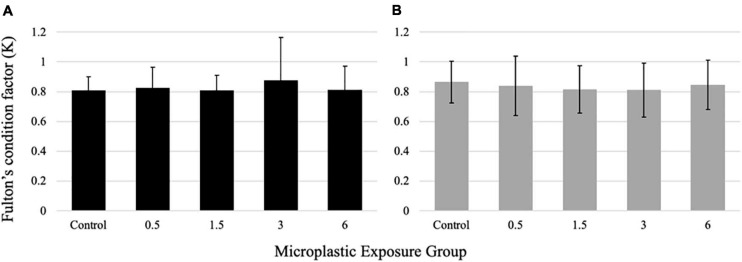
Fulton’s Condition Factor (K) for larval **(A)** and juvenile **(B)** medaka after 21 days of exposure to 0, 0.5,1.5, 3, and 6 fibers/fish per day. Data are displayed as mean ± standard deviation (nested ANOVA, *p < 0.05, *n* = 5 pools of 50 individuals).

All fish weights and lengths (total length) were recorded after sacrifice. No significant differences were found for larvae condition factor between the exposure groups (Figure 7A). Similarly, no differences were seen in the condition factor in the juvenile fish in response to the PE fiber exposure (Figure 7B).

## Discussion

Chronic, low dose exposure to environmentally relevant PE fibers induced subtle changes in larval and juvenile fish health. The reported data indicate that juvenile stages are potentially more susceptible to PE fiber exposure compared to the early larval stage, as fibers were retained significantly longer in the GIT at lower concentrations (3 fibers/fish/day) and, although not statistically significant, digestive gene expression changes were more pronounced.

Polyethylene fiber exposure revealed that microplastic ingestion affects the expression of a key taurine transporter gene, slc6a6. Measurements of ISN, GLP, PYY, and TRP failed to demonstrate any statistically significant difference at the studied concentrations. While these molecular markers, which are involved in glucose metabolism, energy homeostasis, weight gain, and proteolysis, were not differentially expressed in both age groups, the taurine membrane transporter slc6a6 was significantly downregulated in the larvae exposed to the highest microplastic concentration in this study, similar to changes seen in previous studies (Murashita et al., 2009; Polakof et al., 2011; Rønnestad et al., 2013). A similar trend, but not significant, was also observed in juveniles. The primary function of slc6a6 is the transport of taurine across the cellular membrane; therefore, the downstream effect of slc6a6 downregulation is a decreased concentration of intracellular taurine (Hansen et al., 2006; Jong et al., 2012). Taurine is vital in mitochondrial function, which stabilizes mitochondria and prevents excess reactive oxygen species (ROS) leakage (Hansen et al., 2006; Jong et al., 2012; Seidel et al., 2019). Mitochondria have two primary functions, the production of energy and maintenance of cellular metabolism (Seidel et al., 2019). Oxidative stress results from an imbalance of ROS and antioxidants, controlled by mitochondria (Blier, 2014). Slc6a6 is also a solute transporter gene associated with the Nrf2 pathway. The Nrf2 pathway is a “master regulator” in antioxidant response (Hybertson et al., 2011). Since antioxidant pathways counterbalance oxidative stress, a change in the relative expression of key genes in the Nrf2 pathway could indicate oxidative stress (Ma, 2013). Slc6a6 expression is shown to be upregulated with the activation of the Nrf2 pathway indicating the observed downregulation of slc6a6 seen in this study could implicate lower activation of the Nrf2 pathway (Hiebert et al., 2018). Similar results were seen in copepods after ingestion of polystyrene beads resulting in modification of molecular expression of key genes involved in the Nrf2 pathway (Jeong et al., 2017). A study exposing fish kidney leukocytes to virgin microplastics also noted a molecular change in Nrf2 genes (Espinosa et al., 2018). The impact of PE microplastics on bivalves indicated that exposure altered the activity of antioxidant enzymes CAT and GST (Abidli et al., 2021). Similar changes were reported in *Chironomus riparius* (Diptera) larvae after exposure to PE microplastics; ingestion resulted in oxidative damage including the deregulation in antioxidants (Silva et al., 2021). Amphibian (*Physalaemus cuvieri*) exposure to PE also demonstrated increased ROS production and changes to antioxidant metabolism. Higher ROS production indicative of oxidative stress was also observed in human epithelial cells (T98G line) in response to PE microplastic exposure (Schirinzi et al., 2017). These findings collectively indicate antioxidant assessment related to oxidative stress, including enzyme activity and specific pathways like the Nrf2 pathway, are important endpoints for determining the impact of microplastic ingestion at the molecular level (Hybertson et al., 2011; Ma, 2013; Jeong et al., 2017; Abidli et al., 2021; Silva et al., 2021). Slc6a6 is also associated with colorectal adenocarcinomas, which could impact nutrient absorbance and overall fish health (Janikowska et al., 2018). Reduction in taurine could also implicate alteration to mitochondrial activity impacting energy production and metabolism (Hansen et al., 2006; Osellame et al., 2012; Seidel et al., 2019). While no significant differences were seen for the tested genes in juvenile fish, a trend of decreased expression was observed in a non-monotonous dose-response pattern, which was more prevalent than in the larvae (Araujo et al., 2020). However, the inter-individual variance and a relatively low *n*-value led to no statistical significance (Figures 5A2–E2) but indicating a potential threshold concentration between 3 and 6 PE fibers per day. Thus, it is hypothesized that higher exposure concentrations beyond this hormetic range may result in impaired GIT function and subsequently organism health.

Similar to polystyrene microplastic sphere exposure (*O. latipes*, adult and *Oryzias melastigma*, 3-month-old), GIT dysbiosis was observed after exposure to PE fibers at environmentally relevant concentrations comprising the phyla Proteobacteria and Bacteroidetes (Fackelmann and Sommer, 2019; Feng et al., 2020; Huang et al., 2020). PE fiber exposure oppositely modified the Proteobacteria proportion, the most abundant phylum in both larval and juvenile fish GITs. The reduction of proteobacteria in larvae may be attributed to the Xanthobacteraceae, majorly consisting of *Pseudorhodoplanes*, which has been similarly reported in the soil oligochaete *Enchytraeus crypticus* exposed to polystyrene microplastic spheres and been associated with nitrate reduction and nitrogen cycling (Oren, 2014; Zhu et al., 2018). These changes in *Pseudorhodoplanes* abundance were related to worm weight and reproduction changes, indicating reproduction may be a key endpoint for monitoring the effects of microplastic ingestion. However, for juveniles, an increasing abundance shift in Proteobacteria is consistent with other recent studies (Lu et al., 2018). For both, larval and juvenile samples, a trend of abundance increase paralleling the microplastic concentration was seen in Bacteroidetes, and a finding shared with Feng et al. (2020) and Zhu et al. (2018) upon polystyrene sphere exposure in adult medaka and oligochaetes. Bacteroidetes families, specifically Flavobacteriaceae, have been associated with biofilm development on microplastics, thus indicating the shift in microbiota composition observed in these studies may be due to the ingestion of microplastic surface biofilms (Pinto et al., 2019). Both, Proteobacteria and Bacteroidetes are microbial species known to colonize microplastic surfaces (Tu et al., 2020). Microbiota communities of exposed medaka shifting toward phylum profiles associated with biofilms indicate that microplastic ingestion possibly impacts the medaka’s intestinal function in a persistent manner. Shifts in microbial communities toward the order Flavobacteriales have been shown as a progressive oxidative stress response (Lai et al., 2020). While not significant, there is an increasing trend in *Flavobacterium* abundance for the larvae exposed to PE fibers; thus, this could indicate a low chronic oxidative stress level in the organism, further corroborated by an observed oxidative stress response in correlation to polystyrene exposure (Feng et al., 2020). Only few studies have assessed the effects of PE exposure on microbial communities. The results presented here indicated that PE may have impacts similar to those of polystyrene on microbial community composition.

Polyethylene fiber exposure did not result in modifications on the tissue level, like inflammation, microvilli morphology, goblet cell abundance, and mucus pH. Thus, chronic PE fiber consumption at these low doses for 21 days did not induce severe impacts on GIT morphology. Previous research revealed that the size of microplastic and exposure concentration per day play a significant role in the effects of microplastic exposure (Jeong et al., 2017). Thus, it may be carefully concluded that a 4:1 fish:microplastic length ratio, as measured in field samples is not inducing GIT tissue damage. In stark contrast to our results, microplastic exposure in adult medaka resulted in significant impacts at the cellular level after histological evaluation, including inflammation and altered morphology of most gill lamella as well as increased mucous cells and secretion in the foregut; however, that study employed an exposure concentration of 10,000 fibers/L, which is nearly 100 × higher than concentrations used in this study (Hu et al., 2020).

A fish’s energy budget has three significant components: maintenance, somatic growth, and gonad growth. The energy available for growth and reproduction is determined by the energy intake minus the energy used for baseline metabolism and excretion (Forseth et al., 1994). A reduction in energy intake reduces the energy budget available for growth as baseline metabolism and excretion must be maintained to survive. A restructuration of the energy budget has been seen in oysters upon ingestion of polystyrene microbeads at 0.023 mg/L; more energy was allocated to the growth and structure than reproduction (Sussarellu et al., 2016). A study on the effect of microplastic ingestion in clams noted a two-fold reduction in energy intake a resulting in reduced energy availability for maintenance and movement (Xu et al., 2017). The PE fiber exposure in the present study did not significantly impact the larvae or juvenile energy budget, as fish growth, an apical endpoint and a significant fish health indicator, was not affected. Literature reports are contradictory regarding microplastic effects on the fish condition. While some studies indicate that microplastic ingestion can impact growth and survivability, others reported the absence of whole-organism growth effects, similar to the here reported results (Jeong et al., 2017; Karami et al., 2017; Choi et al., 2018; Jovanovic et al., 2018; Weber et al., 2018; Hu et al., 2020). A study exposing seabream to six different types of microplastics, including PE, observed no significant alteration to the exposed fish’s growth rate (Jovanovic et al., 2018). Previous research on zebrafish exposed to low-density PE noted similarly no significant changes in exposed fish’s condition factor (Karami et al., 2017). However, a study assessing the impacts of both virgin PE and PE harbored from the environment on glassfish reported a decrease in overall fish size after exposure to microplastics for 3 months at a concentration of 0.010 g/20L (Naidoo and Glassom, 2019). The discrepancy among research observations is likely due to the variation of fish species, plastic polymers, sizes of microplastics, exposure duration, and concentrations utilized. Previous research involving different sizes and shapes of microplastics has identified these factors to be crucial in determining the impact of microplastic exposure (Jeong et al., 2017; Jabeen et al., 2018).

Based on this study’s findings, there is no imminent impact of microplastic exposure on fish health at microplastic concentrations resembling the present average concentrations being ingested by larval and juvenile fish in the field (Lusher et al., 2013; Lenz et al., 2016; Beer et al., 2018). No organism level consequences were observed after exposure to PE in the present study. However, it is noted that both larval and juvenile medaka demonstrated slight molecular alterations which may be regarded as an early warning sign for potentially more severe oxidative stress damage as seen in other studies which employed higher microplastic exposure concentrations. Notwithstanding the lack of immediate, severe consequences observed in this study, the results hint toward an increased need of monitoring microplastic levels in vital spawning and nursery grounds for commercially important fish as both the larval and juvenile stages could be even more susceptible to chronic exposure and possible long-term effects.

## Conclusion

Several studies have reported significant adverse effects of microplastic consumption in fish. This study examined the effect of microplastic ingestion on gene expression, gut microbiota, GIT integrity, and fish growth and condition during two early life history stages. Our results indicate PE fibers pose little imminent threat to fish health at environmentally relevant concentrations when examining growth and condition. However, this study observed potential disruption to the energy intake at the highest concentration of exposed larvae based on gene expression and microbiota composition results. While not all significant, changes in microbial communities did show a shift in microbiota composition similar to previous studies, which indicate a stress response. While the concentrations of microplastics used in this study did not cause significant alterations to the overall microbiota communities, the trend of the results demonstrated there could be significant impacts of microplastic ingestion on GIT microbiota communities, potentially leading to long-term changes in digestive function and metabolism of exposed fish. Further investigation of the molecular effects of microplastic ingestion on key genes related to the Nrf2 pathway could provide more evidence supporting the conclusion that microplastic ingestion impacts fish energy metabolism. Expanding investigated life stages to adults would allow further investigation of potential reproductive effects, including gonad morphology and selected reproductive gene expression, and may deliver more insight into the underlying mechanisms. Assessing the exposed fish’s chemical body burden would provide evidence of any potential endocrine-disrupting chemicals leakage from the administered PE fibers. Future research on the effects of common microplastics, both virgin and UV weathered, could provide more insight into microplastics’ effects in the environment impacting wild fish. Specifically, a study targeting antioxidant responses would provide more direct assessment of potential effects of microplastic consumption on oxidative stress in fish given the data provided here. Future studies involving similar relevant concentrations could provide vital evidence on the current threat microplastic pollution poses to wild fish populations.

## Data Availability Statement

The raw data supporting the conclusions of this article will be made available by the authors, without undue reservation. The 16S rRNA gene sequences generated for this study can be found through BioProject PRJNA748405 at the NCBI’s Sequence Read Archive. Other raw data can be found in a GitHub repository linked in the Supplementary Material.

## Ethics Statement

The animal study was reviewed and approved by Texas A&M University-Corpus Christi Institutional Animal Care and Use Committee # 05-19.

## Author Contributions

ED performed experiments, data analysis, and drafting of the manuscript. LP conducted the microbiota sequencing, figure design, and data analysis. AH-H developed methodological procedures and assisted with manuscript draft. JT contributed to the conception of microbiota analysis and provided supervision as well as feedback on the manuscript draft. SG contributed experimental design and theoretical framework of the research and feedback on manuscript draft. FS provided over all experimental design and research concept, data analysis, and the manuscript draft conceptualization. All authors contributed to the article and approved the submitted version.

## Conflict of Interest

The authors declare that the research was conducted in the absence of any commercial or financial relationships that could be construed as a potential conflict of interest.

## Publisher’s Note

All claims expressed in this article are solely those of the authors and do not necessarily represent those of their affiliated organizations, or those of the publisher, the editors and the reviewers. Any product that may be evaluated in this article, or claim that may be made by its manufacturer, is not guaranteed or endorsed by the publisher.
