# Supplementary material for: A Holistic Assessment of Polyethylene Fiber Ingestion in Larval and Juvenile Japanese Medaka Fish
Source: Front Physiol. 2021 Aug 4;12:668645. doi: 10.3389/fphys.2021.668645 (PMC8371532; doi:10.3389/fphys.2021.668645)
Supplement: Supplementary file 1 [file Data_Sheet_1.PDF]

Supplementary Material

1 Supplementary Table

Supplementary Table 1. List of *Oryzias latipes* primers used for RT-qPCR assessment.

Primer Pair Name	Prime Pair Sequence (5’ to 3’)	Primer Pair Function	Accession #
OL glucagon-1-like F1<br><br>*OL* glucagon-1-like R1	AGCGACGTGAACAAAGTCCT<br><br>CGTCGAAACAAGCAGATCAA	Amplification of digestive hormone gene glucagon- like peptide 1	XM_011483973.3
OL peptide YY-like F1<br><br>*OL* peptide YY-like R1	CCTGAGGAGCTTGCCAAATA<br><br>AGGCAGACCCTCATACCTGAT	Amplification of digestive hormone gene peptide YY-like	XM_020710138.2
OL slc6a6 F1<br><br>*OL* slc6a6 R1	TACTCTGCATGGGCTGCTTTA<br><br>TCAGAGGCCCTTCAGACTGT	Amplification of nutrient membrane transporter solute carrier family 6 member 6	XM_004070517.4
OL trypsinogen F1<br><br>*OL* trypsinogen R1	ACTCAGCAGCCATGAGGTCT<br><br>TCCACTCGGGACTGGTAGC	Amplification of digestive enzyme gene trypsinogen	AB272106.1, NM_001104900.1
OL insulin F1<br><br>*OL* insulin R1	CAGCAGAACCTCCTCTGGTC<br><br>GAGGGTCCACGTCTCTCTTG	Amplification of peptide hormone insulin	XM_004084322.3
OL 18S F1<br><br>*OL* 18S R1	GGCCGTTCTTAGTTGGTGGA<br><br>CCCGGACATCTAAGGGCATC	Amplification of 18S reference gene	XR_002874070.1, AB105163.1

OL EF1 alpha F1<br><br>*OL* EF1 alpha R1	TTCAAGGGTGAGGTCAAGGC<br><br>ATAACTCTCTTGGCACCGCC	Amplification of elongation factor 1 alpha reference gene	NM_001104662.1
OL RPL7 F1<br><br>*OL* RPL7 R1	T CTGCCTGTACTCCTTGTGGT<br>CTTTCTCGGTGGTCGCAAT	Amplification of ribosomal protein L7 reference gene	NM_001104870

2 Supplementary Figures

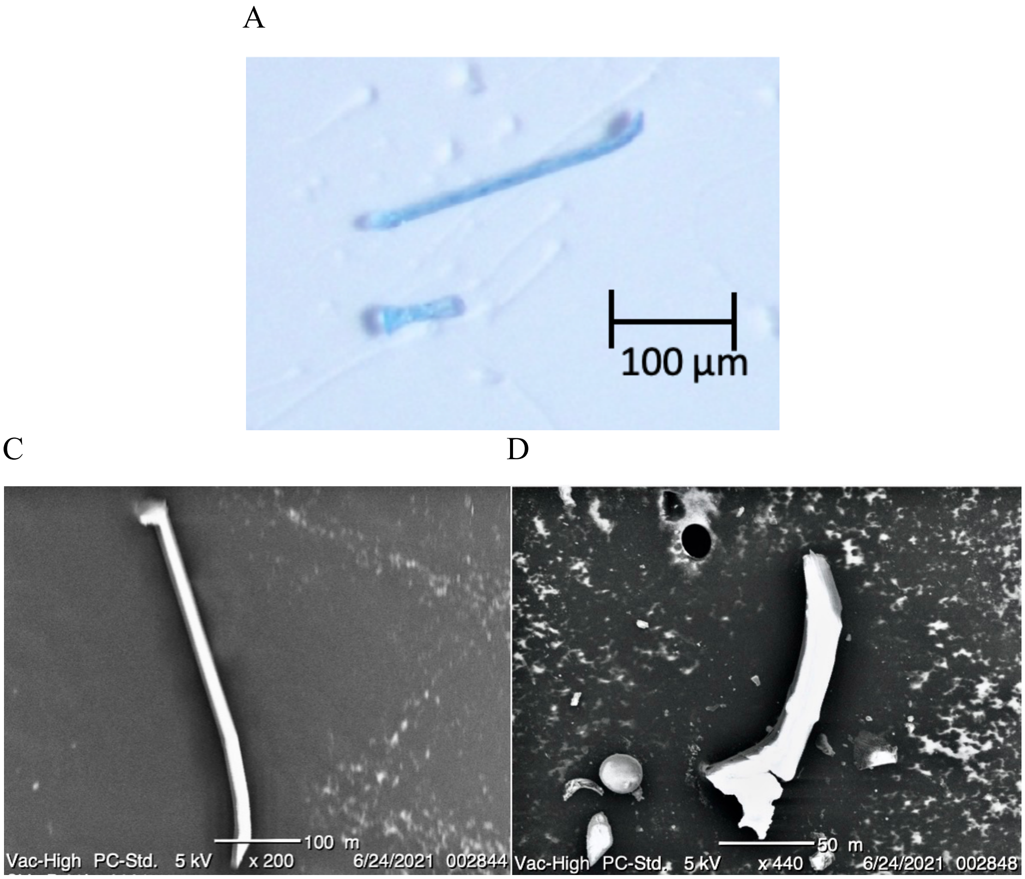

Supplementary Figure 1. Images of blue, low-density polyethylene microplastic fibers used in exposure. (A) Dissecting scope image of both 100 µm and 400 µm fivers; larval life stage received 100 µm and juvenile life stage received 400 µm. (B) Scanning Electron Microscope (SEM) image of 100 µm PE fiber. (C) SEM image of 400 µm PE fiber.

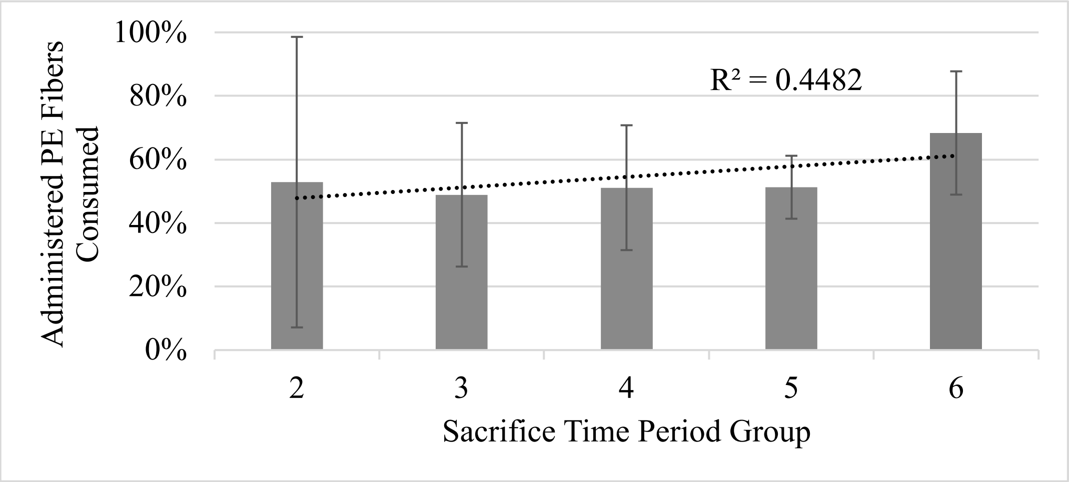

Supplementary Figure 2. Consumption of 100 µm PE fibers by *O. latipes* larvae after 1 hour of feeding time. *O. latipes* larvae readily consumed at least 50% of fibers administered (n=9). Linear trendline with R-squared value of 0.4482 displayed. Error bars displayed are mean ± sd.

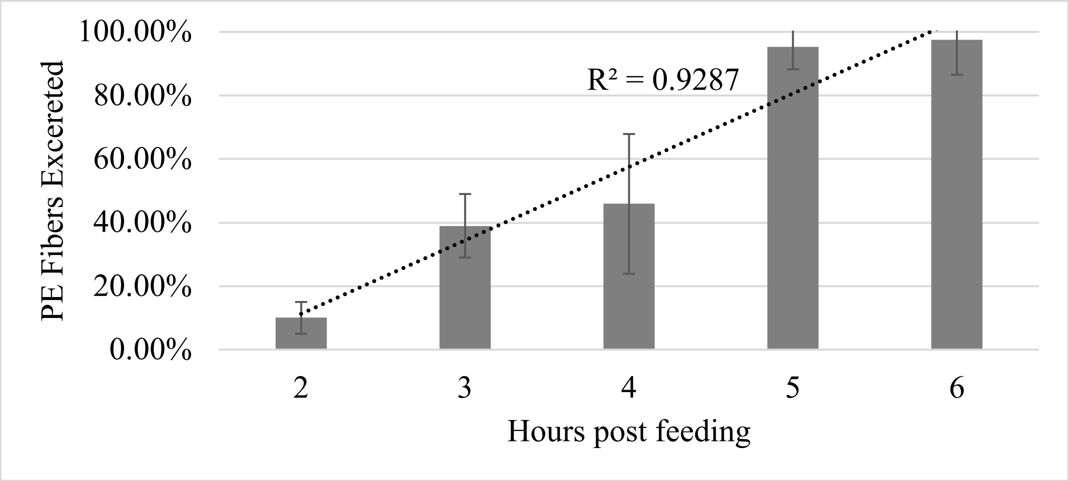

Supplementary Figure 3. Percent of PE fibers excreted by *O. latipes* larvae 2, 3, 4, 5 and 6 hours post feeding. Linear trendline with R-squared value of 0.9287 displayed (n=9). Error bars displayed are mean ± sd.

A

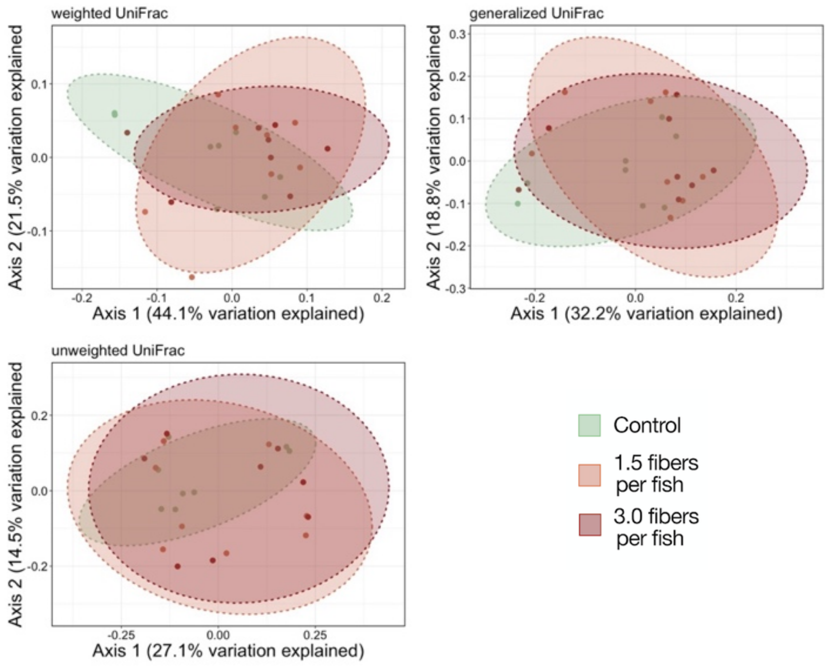

B

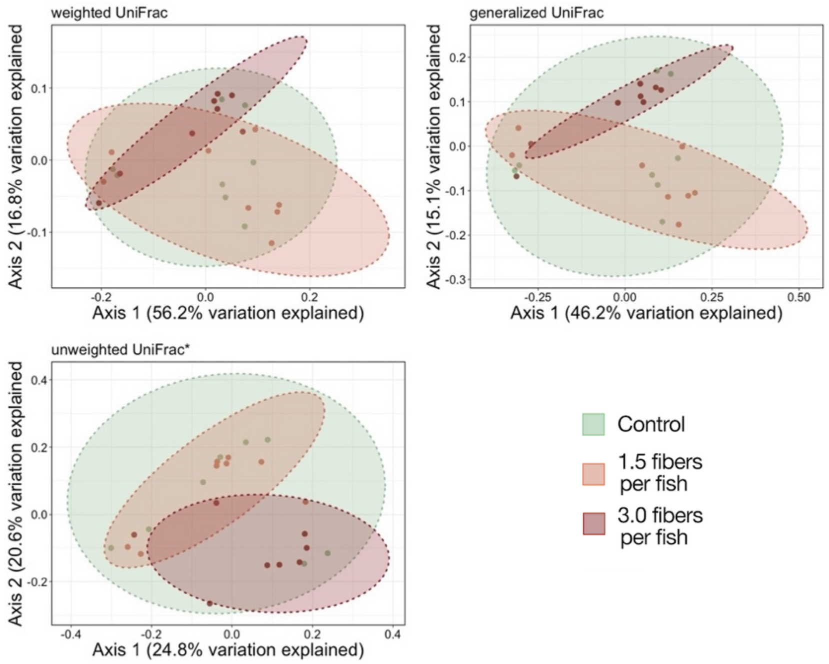

Supplementary Figure 4. (A) PCoA of gut microbial communities from larval fish, Samples are colored by exposure concentration. Differences between age groups were not significant using any of the three UniFrac values (PERMANOVA, $p > 0.05$). (B) PCoA of gut microbial communities from juvenile fish. Samples are colored by exposure concentration. The only significant difference was between 1.5 fibers/fish/day and 3 fibers/fish/day using unweighted UniFrac distances (PERMANOVA, $p > 0.05$).

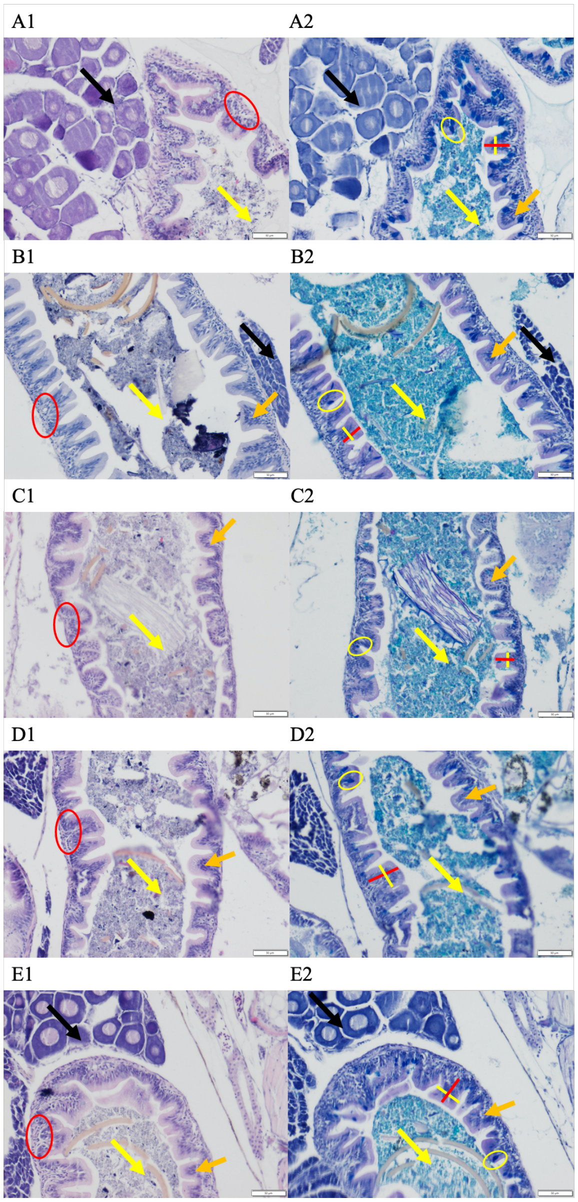

Supplementary Figure 5. Histological sections of larval hindgut from each exposure concentration. (A) Histological section of larval *OL* from control (0 fibers/fish/day) stained in H&E stain (A1) and AB-PAS stain (A2). (B) Histological section of larval *OL* from 0.5 fibers/fish/day stained in H&E stain (B1) and AB-PAS stain (B2). (C) Histological section of larval *OL* from 1.5 fibers/fish/day stained in H&E stain (C1) and AB-PAS stain (C2). (D) Histological section of larval *OL* from 3 fibers/fish/day stained in H&E stain (D1) and AB-PAS stain (D2). (E) Histological section of larval *OL* from control 6 fibers/fish/day stained in H&E stain (E1) and AB-PAS stain (E2). No significant differences were found in the exposure concentrations when compared to the control for the indices measured (goblet cell count, microvilli width/length, mucus pH, and leukocyte infiltration). Black arrows indicate gonadal tissue, yellow arrows indicate gut lumen contents, orange arrows indicate microvilli, red circles encompass example area assessed for leukocyte infiltration, yellow circles encompass goblet cells, lines indicate example microvilli measurements, length (red) and width (yellow).

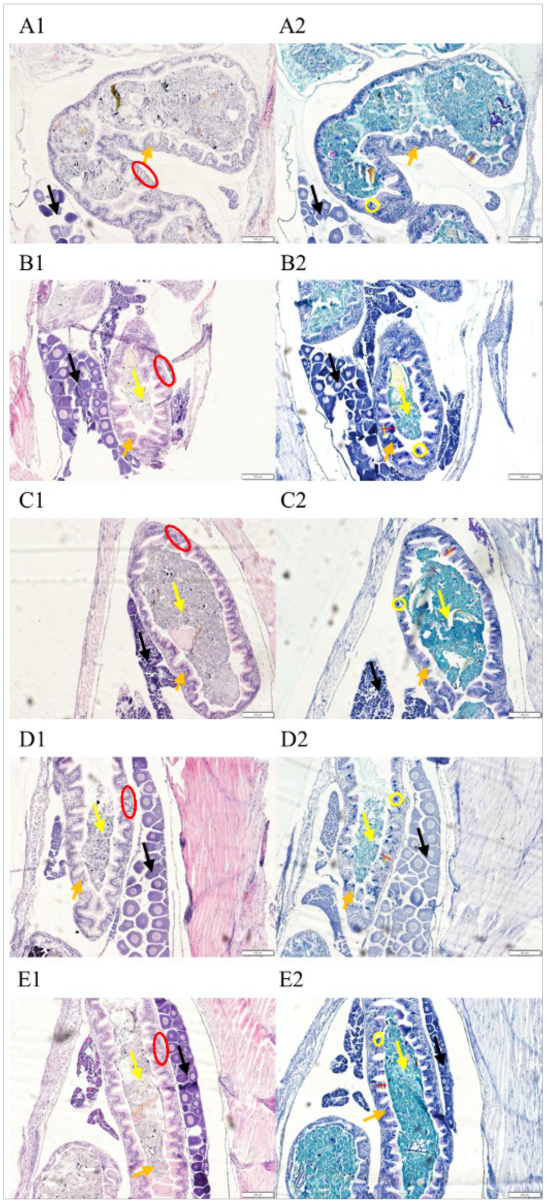

Supplementary Figure 6. Histological sections of juvenile hindgut from each exposure concentration. (A) Histological section of juvenile *OL* from control (0 fibers/fish/day) stained in H&E stain (A1) and AB-PAS stain (A2). (B) Histological section of juvenile *OL* from 0.5 fibers/fish/day stained in H&E stain (B1) and AB-PAS stain (B2). (C) Histological section of juvenile *OL* from 1.5 fibers/fish/day stained in H&E stain (C1) and AB-PAS stain (C2). (D) Histological section of juvenile *OL* from 3 fibers/fish/day stained in H&E stain (D1) and AB-PAS stain (D2). (E) Histological section of juvenile *OL* from control 6 fibers/fish/day stained in H&E stain (E1) and AB-PAS stain (E2). No significant differences were found in the exposure concentrations when compared to the control for the indices measured (goblet cell count, microvilli width/length, mucus pH, and leukocyte infiltration). Black arrows indicate gonadal tissue, yellow arrows indicate gut lumen contents, orange arrows indicate microvilli, red circles encompass example area assessed for leukocyte infiltration, yellow circles encompass goblet cells, lines indicate example microvilli measurements, length (red) and width (yellow).

3 Supplementary Data Files

https://github.com/edibona1/PE-fiber-ingestion-in-fish
